# Knowledge Domains and Emerging Trends of Osteoblasts-Osteoclasts in Bone Disease From 2002 to 2021: A Bibliometrics Analysis and Visualization Study

**DOI:** 10.3389/fendo.2022.922070

**Published:** 2022-07-22

**Authors:** Jun Hou, Hongjie Su, Xiaocong Kuang, Wencong Qin, Kaibing Liu, Kaixiang Pan, Bokai Zhang, Sijie Yang, Shenghui Yang, Xiao Peng, Xinyu Nie, Qikai Hua

**Affiliations:** ^1^ Department of Bone and Joint Surgery, Research Centre for Regenerative Medicine, The First Affiliated Hospital of Guangxi Medical University, Nanning, China; ^2^ Guangxi Diabetic Foot Salvage Engineering Research Center, Guangxi Medical University, Nanning, China; ^3^ Department of Orthopaedics, The Second Hospital, Jilin University, Changchun, China

**Keywords:** osteoblasts, osteoclasts, bone diseases, bibliometrics, citespace, vosviewer

## Abstract

**Background:**

Osteoblasts-Osteoclasts has been a major area in bone disease research for a long time. However, there are few systematic studies in this field using bibliometric analysis. We aimed to perform a bibliometric analysis and visualization study to determine hotspots and trends of osteoblasts-osteoclasts in bone diseases, identify collaboration and influence among authors, countries, institutions, and journals, and assess the knowledge base to develop basic and clinical research in the future.

**Methods:**

We collected articles and reviews for osteoblasts-osteoclasts in bone diseases from the Web of Science Core Collection. In addition, we utilized scientometrics software (CiteSpace5.8 and VOSviewer1.6.18) for visual analysis of countries/regions, institutions, authors, references, and keywords in the field.

**Results:**

In total, 16,832 authors from 579 institutions in 73 countries/regions have published 3,490 papers in 928 academic journals. The literature in this field is rapidly increasing, with *Bone* publishing the most articles, whereas J*ournal of Bone and Mineral Research* had the most co-cited journals. These two journals mainly focused on molecular biology and the clinical medicine domain. The countries with the highest number of publications were the US and China, and the University of Arkansas for Medical Sciences was the most active institution. Regarding authors, Stavros C. Manolagas published the most articles, and Hiroshi Takayanagi had the most co-cited papers. Research in this field mainly includes molecular expression and regulatory mechanisms, differentiation, osteoprotection, inflammation, and tumors. The latest research hotspots are oxidative stress, mutation, osteocyte formation and absorption, bone metabolism, tumor therapy, and in-depth mechanisms.

**Conclusion:**

We identified the research hotspots and development process of osteoblasts-osteoclasts in bone disease using bibliometric and visual methods. Osteoblasts-osteoclasts have attracted increasing attention in bone disease. This study will provide a valuable reference for researchers concerned with osteoblasts-osteoclasts in bone diseases.

## Introduction

Bone tissue is a hard connective tissue consisting of cells, fibers, and a matrix. Under normal circumstances, bone tissue remains in a steady state while undergoing a series of continuous shaping and repair processes to maintain the dynamic balance (1). Among them, osteoblasts are bone-forming cells that can synthesize the bone matrix, regulate mineralization, and eventually differentiate into bone cells or bone coating cells. Osteoclasts are cells responsible for bone resorption and play an important role in bone formation and regulation of bone density. Osteoclasts in bone absorption and osteoblasts in bone formation play the role of mechanical sensor and coordinator in the bone reconstruction process, respectively, in the presence of local (e.g., growth, cytokines, and inflammatory factors) and systemic factors (e.g., calcitonin and estrogen). The control of these factors promotes bone homeostasis (2–4). Evidence has shown that osteoclasts play a critical role in many diseases such as osteoporosis, myeloma, rheumatoid arthritis, osteosarcoma, osteosclerosis, and Paget’s disease of bone (5–8).

Owing to the strong potential of osteoblasts-osteoclasts in bone homeostasis and bone remodeling, a rapid increase in the literature has occurred in recent years. Many researchers have reviewed osteoblasts-osteoclasts in bone diseases from multiple aspects. For example, Koichi Matsuo et al (9). reviewed the communication between osteoblasts and osteoclasts in the different stages of bone remodeling and summarized bone remodeling into three stages: initiation, transformation, and termination. The initial stage includes chemokines, receptor activator of nuclear factor κB ligand (RANKL), and stimulus molecules. The transition stage contains coupling factors and membrane-bound molecules. Finally, the termination stage covers osteoprotegerin (OPG) production and WNT and RANKL signaling pathways. Xiao Chen et al (4) reviewed the interaction between osteoblasts and osteoclasts, including the formation and apoptosis of osteoclasts induced by osteoblasts and the role of osteoclasts in bone formation. They described important early studies such as those on OPG, RANKL, and Ephrin2/EphB4. T.C.A. Phan et al (8). described osteogenesis and bone formation and osteoclast and bone resorption and reviewed the role of osteoblasts-osteoclast in bone diseases, including osteoporosis, osteosclerosis, osteogenesis imperfecta, periodontitis, osteoarthritis, Paget’s disease of bone, aseptic loosening.

At present, many methods systematically evaluate a research field, among which bibliometrics is one of the most used. Literature metrology is an applied mathematics and statistics method used to investigate the discipline of books and other media (10). It can be used not only for qualitative and quantitative analysis in a study in the field of authors, institutions, countries, and regions, as well as co-cited authors, journals, and references, but it can also help researchers quickly grasp the research hotspots and development trends of a particular field. This cannot be achieved by other methods such as traditional review, meta-analysis, or experimental research. In recent years, bibliometrics analysis has received increasing attention (7, 11–14). Owing to its powerful analysis and visualization capabilities, bibliometrics is suitable for the evaluation and review of osteoblasts-osteoclasts in bone diseases.

Using CiteSpace and VOSviewer, two commonly used bibliometric tools, the purpose of this study was to draw knowledge maps through their powerful network cooperative analysis and discuss the research hotspots and development trends of osteoblasts-osteoclasts in bone diseases over the past 20 years by performing the following steps. First, we analyzed the authors, institutions, countries/regions, and journals, co-cited authors, journals, and literature to obtain the most relevant general information and cooperation information in this field. Second, the most concentrated literature and the turning point of new research directions were analyzed in this field *via* the co-cited references. Third, the knowledge structure and hotspot evolution in the research field of osteoblasts-osteoclasts in bone disease were determined through a burst analysis of keywords and co-citation literature, providing new ideas for basic research and clinical application.

## Materials and Methods

### Data Collection

**Figure 1:**
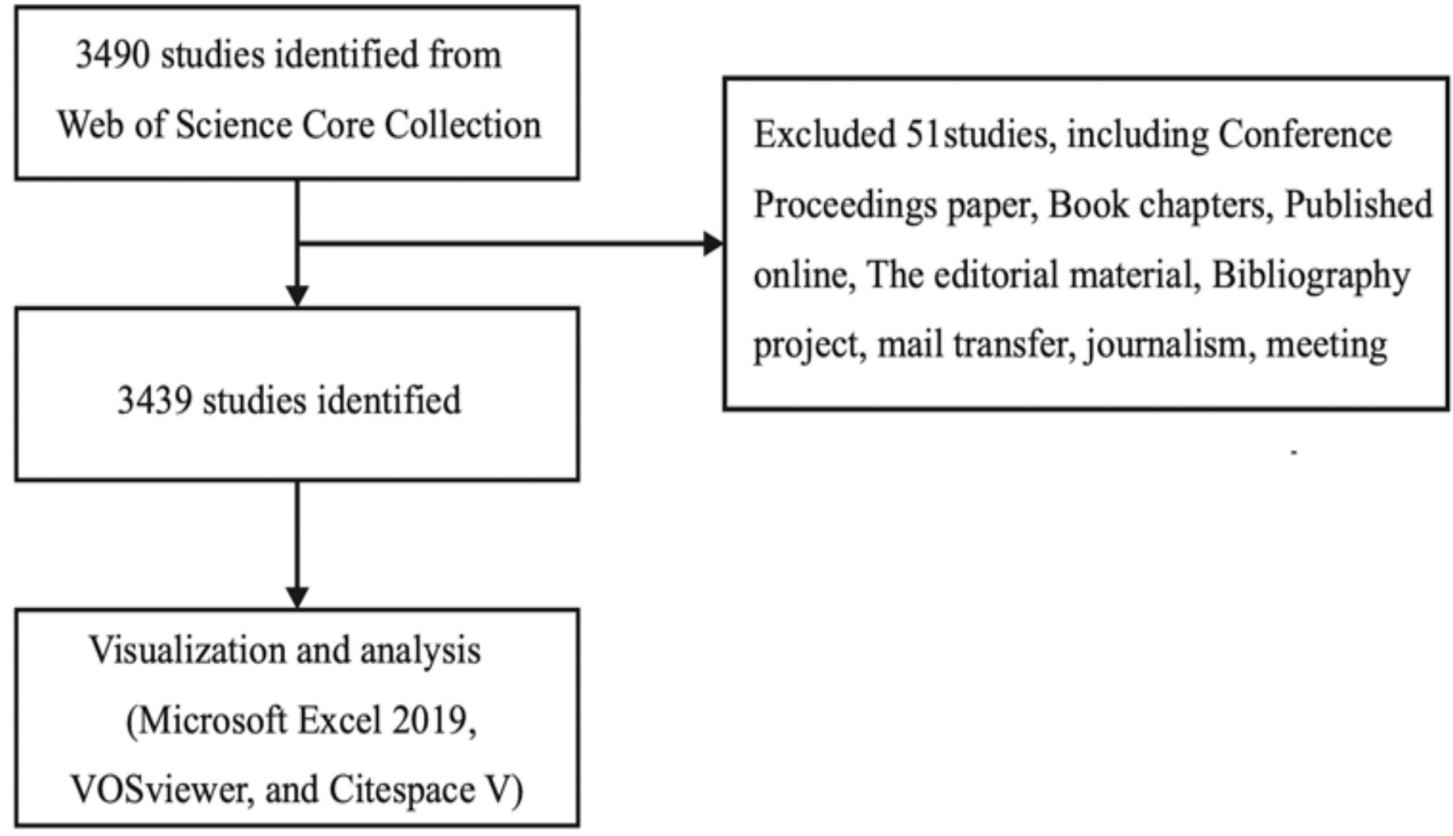
Flow chart of literature screening.

The data analyzed in bibliometrics were downloaded from the WOS database. This database was selected because it currently contains more than 12,400 authoritative and high-impact academic journals worldwide, and thus is regarded as the most influential database that can provide the comprehensive data information required by bibliometrics software (12, 15–17). The advanced search entry in the database was set as “osteoblast & osteoclast & bone diseases,” the considered period went from January 1, 2002, to December 31, 2021, and selected papers and review papers were downloaded on February 2, 2022. Proceedings papers, conference abstracts, book chapters, online publications, editorial material, revisions, bibliographic items, and letters were excluded. A total of 3,490 articles were retrieved, of which 51 articles were excluded and the remaining 3,439 articles were exported as plain text, and the file was named “download_txt” (**Figure 1**).

### Data Analysis and Visualization

At present, the most commonly used bibliometrics software include CiteSpace, VOSviewer, CitNetExplorer, Sci2 TOOL, Pajek, and Gephi. Among them, Pajek and Gephi are the most powerful in network analysis and network visualization, respectively. However, CiteSpace was selected owing to its wide range of functions, which not only include network analysis and network visualization but also turning points, clustering automatic naming, macro theory, double graph superposition, concept tree, and timeline functions. As the heat map function of VOSviewer is not available in other analysis software, this software (VOS viewer 1.6.18) was selected, along with CiteSpace 5.8, for visual analysis and knowledge mapping, respectively (12, 17, 18).

CiteSpace is an information visualization software based on the citation analysis theory developed by Professor Chaomei Chao, an internationally renowned information visualization expert, using Java language (19). It allows researchers to find the most relevant topics and scientific literature in their knowledge fields, understand the most important and key effective information, clarify their development process, and identify current research frontiers and development trends (20). The outstanding feature of CiteSpace is that it uses a diversified, time-sharing, and dynamic visualization language for citation analysis to display the evolution of the field on a knowledge map of citation networks through an ingenious spatial layout (21). The research frontiers represented by the citation node literature and co-citation clustering as the knowledge base on the atlas are automatically identified, showing the decipherability of the atlas itself. Therefore, we used CiteSpace 5.8 to analyze and visualize the research hotspots and evolution of osteoblasts-osteoclasts in bone diseases over the past 20 years and predict the future development trends in this field.

VOSviewer is a software program developed at Leiden University in the Netherlands for creating, visualizing, and exploring maps based on web data (22, 23). VOSviewer can analyze network data, bibliometric data, and more. It can also be used to construct authors and co-cited authors for free, based on co-cited data, or build keyword maps based on co-occurrence data. The software presents bibliometric maps in different ways, including network, overlay, and density maps, each focusing on different aspects (22). First, we used VOSviewer 1.6.15 to analyze major journals, co-cited journals, authors, co-cited authors, and keyword co-occurrence according to the data from WOS and created the related network and density graphs. In addition, we also produced an overlapping network graph of authors. Through these relevant analyses, we determined the scientific research structure, research hotspots, and development trends in this field.

## Results

A total of 3,490 articles were retrieved, of which 51 were excluded, leaving a total of 3,439 articles to be exported as plain text.

### Trends of Publications in the Past 20 Years

**Figure 2:**
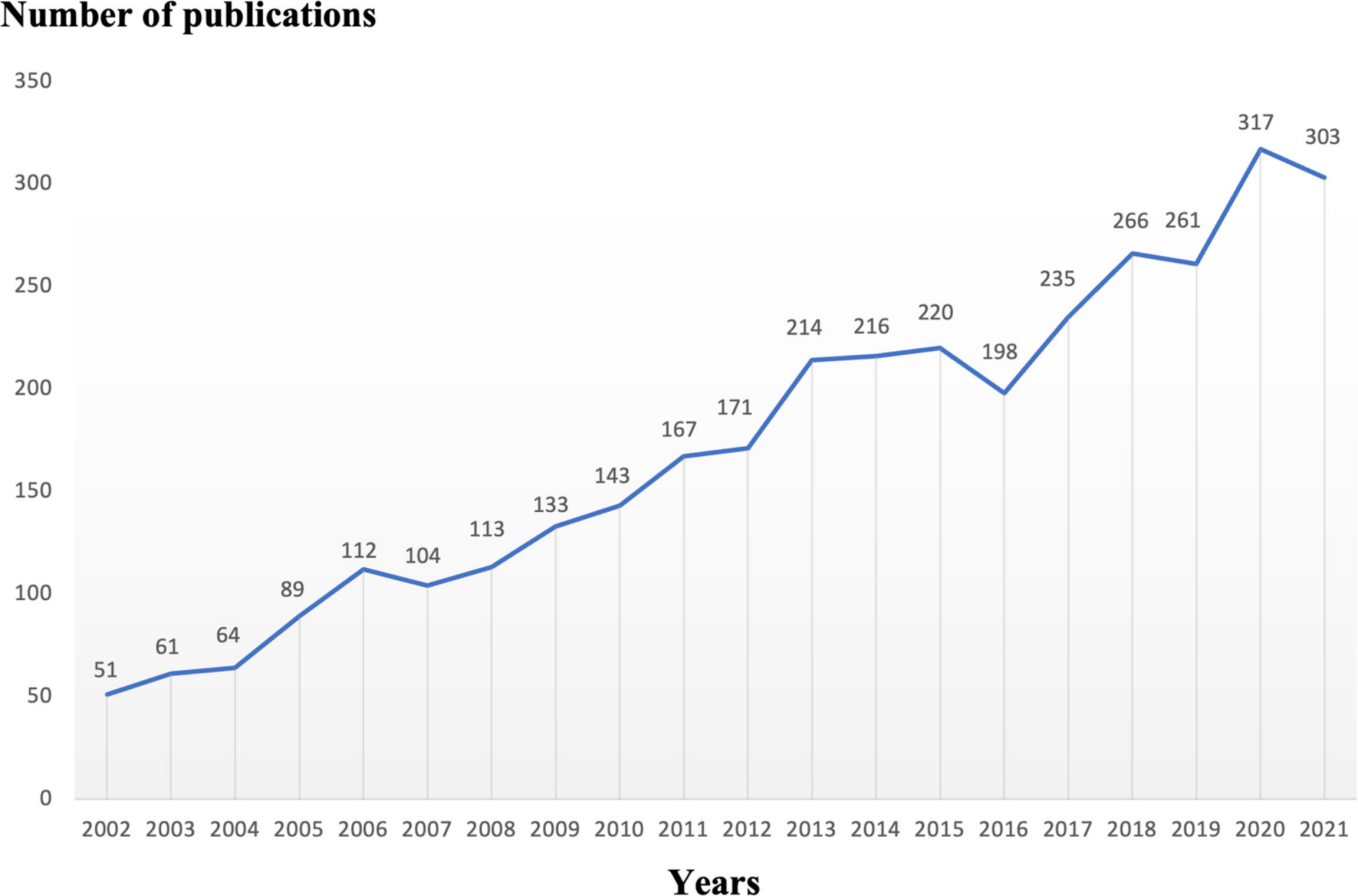
Trends of osteoblasts-osteoclasts published in bone diseases research over the past 20 years.

In a specific research field, the number of articles published in each period determines the trend of a certain research hotspot. For the period from 2002 to 2021, we collected a total of 3,490 eligible articles and concluded with a total of 3,439 related papers and review papers (**Figure 2**). As shown in **Figure 2**, the number of articles related to osteoblasts-osteoclasts in bone diseases shows an increasing trend, indicating that this research topic has been attracting considerable attention. In particular, the number of articles published reached 317 in 2020.

### Major Journals and Co-cited Journals

We used VOSviewer to analyze the journals and co-cited journals publishing the articles, and the results present the most influential journals related to osteoblasts-osteoclasts in bone diseases in the WOS database (**Table 1**). As shown in **Table 1**, 3,439 articles were published in 928 journals, of which *Bone* magazine has the highest rate of publications, followed by *Journal of Bone and Mineral Research*, *Nature Medicine*, *International Journal of Molecular Sciences*, and *Journal of Biological Chemistry.* Among the top 10 journals, five belong to JCR Q1, and eight journals have an impact factor (IF) of more than 5.

**Table 1:** The top 10 journals of osteoblasts-osteoclasts in bone disease research.

Rank	Journal	Count(%)	IF(2021)	JCR
1	Bone	180(4.58%)	4.398	Q1
2	Journal of Bone and Mineral Research	147(4.27%)	6.741	Q1
3	International Journal of Molecular Sciences	86(2.50%)	5.923	Q2
4	Plos One	73(2.12%)	3.24	Q2
5	Scientific Reports	50(1.45%)	4.379	Q1
6	Biochemical and Biophysical Research Communications	49(1.42%)	3.575	Q2
7	Journal of Biological Chemistry	48(1.40%)	5.157	Q2
8	Journal of Cellular Biochemistry	47(1.37%)	4.429	Q2
9	Calcified Tissue International	46(1.34%)	4.333	Q2
10	Journal of Cellular Physiology	45(1.31%)	6.384	Q2

In the analysis of co-cited journals, the results showed that among 8,733 co-cited journals, 25 journals were co-cited more than 1,200 times (**Table 2**). As shown in **Table 2**, the *Journal of Bone and Mineral Research* has the most co-cited occurrences. *Bone*, *Journal of Biological Chemistry*, *Journal of Clinical Investigation*, and *Proceedings of The National Academy of Sciences of The US of America* were among the top ten co-cited journals, of which seven had an IF greater than 5. Among these, two journals exceeded 40, and six belonged to JCR Q1.

**Table 2:** The top 10 co-cited journals of osteoblasts-osteoclasts in bone disease research.

Rank	Co-cited Journal	Count(%)	IF(2021)	JCR
1	Journal of Bone and Mineral Research	2770(2.73%)	6.741	Q1
2	Bone	2526(2.49%)	4.398	Q1
3	Journal of Biological Chemistry	2240(2.21%)	5.157	Q2
4	Journal of Clinical Investigation	1926(1.90%)	14.808	Q1
5	Proceedings of The National Academy of Sciences of The United States of America	1889(1.86%)	11.205	Q1
6	Nature	1775(1.75%)	49.962	Q1
7	Endocrinology	1607(1.58%)	4.736	Q2
8	Cell	1567(1.55%)	41.582	Q1
9	Biochemical and Biophysical Research Communications	1529(1.51%)	3.575	Q2
10	Calcified Tissue International	1312(1.29%)	4.333	Q2

**Figure 3:**
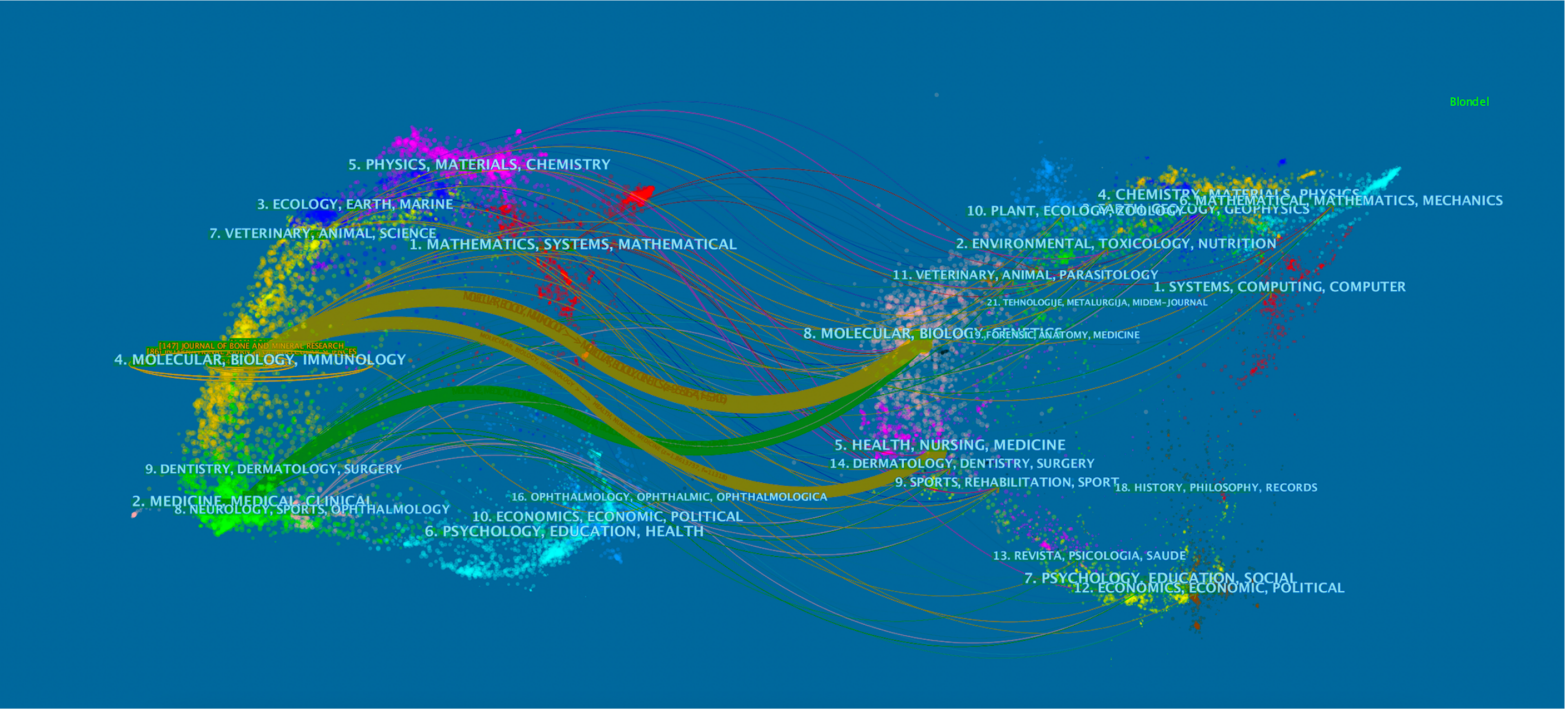
Dual-map overlay of journals related to osteoblasts-osteoclasts in bone disease. Notes: The citing journals are on the left, the cited journals are on the right, and the colored path represents a citation relationship.

As shown in **Figure 3**, the citing journal is on the left, whereas the cited journal is on the right. Paths with different colors indicate the cited relationship. Two yellow and one green citation paths are mainly identified, suggesting that studies published in Molecular/Biology/Genetics journals were mainly cited by studies published in Molecular/Biology/Immunology and Medicine/Medical/Clinical journals, whereas the documents published in Health/Nursing/Medicine journals were often cited by Molecular/Biology/Immunology journals.

### Distribution Countries/Regions and Institutions

**Figure 4:**
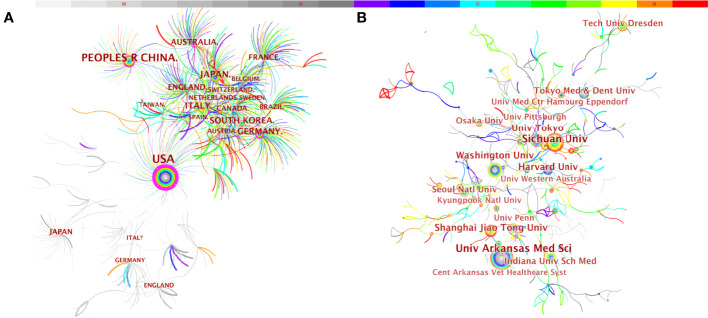
Distribution of publications from different countries/regions **(A)** and institutions **(B)** in osteoblasts-osteoclasts in bone disease. Notes: The size of the node reflects the co-occurrence frequencies, and the links indicate the co-occurrence relationships. The color of node and line represent different years; colors vary from gray to red as time passes from 2002 to 2021.

We used CiteSpace to analyze countries/regions and institutions, and the results showed that a total of 579 institutions from 73 countries/regions jointly published 3,439 articles (**Table 3**). Among the top 10 countries, the US had the largest number of articles (1,132, 24.76%), followed by China (738, 16.15%), Japan (375, 8.20%), Germany (246, 5.38%), South Korea (230, 5.03%), Italy (214, 4.68%), and England (188, 4.11%) (**Figure 4A**). Tree ring history represents the record of articles published in a certain country; different colors of the tree ring represent the corresponding time, and the overall size of the tree ring reflects the number of articles published by countries such as the US, China, Germany, England, Australia, and France, which are characterized by a high centrality (≥ 0.1). The purple outer ring, shown in **Figure 4A**, is often considered an important turning point leading to a transformative discovery (15, 20). Furthermore, the color of tree rings showed that the US (2002), Japan (2002), England (2002), Italy (2002), and Australia (2003) were the first countries to begin research of osteoblasts-osteoclasts in bone disease.

**Table 3:** The top 10 countries/regions involved in osteoblasts-osteoclasts in bone disease research.

Rank	Country/region	Year	N(%)	Centrality
1	USA	2002	1132(24.76%)	1
2	PEOPLES R CHINA.	2008	738(16.15%)	0.22
3	JAPAN.	2007	375(8.20%)	0.08
4	GERMANY.	2008	246(5.38%)	0.24
5	SOUTH KOREA.	2008	230(5.03%)	0.02
6	ITALY.	2008	214(4.68%)	0.04
7	ENGLAND.	2007	188(4.11%)	0.17
8	AUSTRALIA.	2008	159(3.48%)	0.15
9	FRANCE.	2008	135(2.95%)	0.19
10	CANADA	2002	97(2.12%)	0.09

Meanwhile, we also observed the institutional collaboration network on the topic of osteoblasts-osteoclasts in bone diseases (**Figure 4B**). The figure shows that the top five institutions were the University of Arkansas for Medical Sciences (99), Sichuan University (95), Washington University (90), Harvard University (87), and The University of Tokyo (54).

### Authors and Co-cited Authors

**Figure 5:**
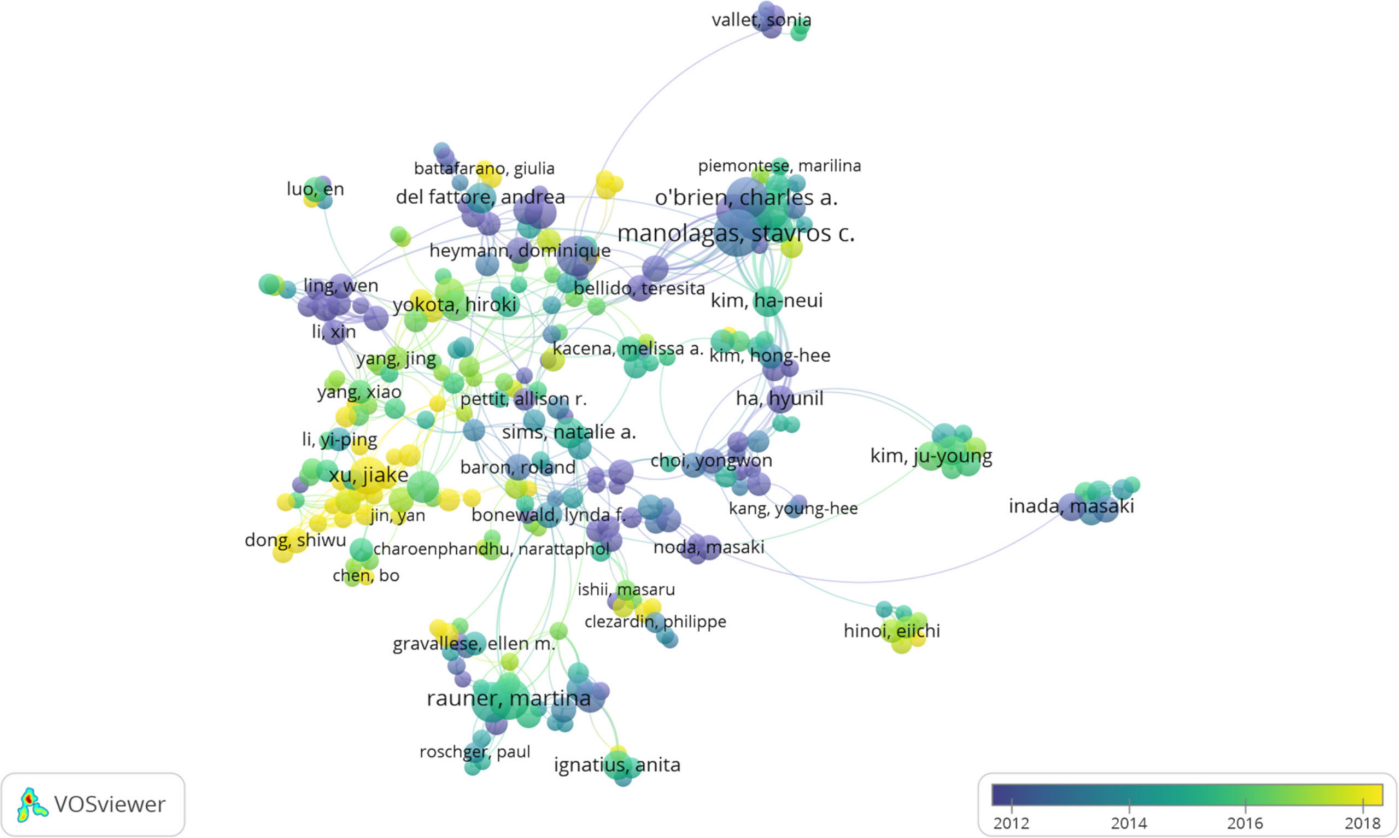
The co-occurrence map of authors in osteoblasts-osteoclasts in bone disease (T≥5). Notes: The size of node reflects the author’s co-occurrence frequencies, the link indicates the co-occurrence relationship between authors, and different node colors indicate different clusters.

**Figure 6:**
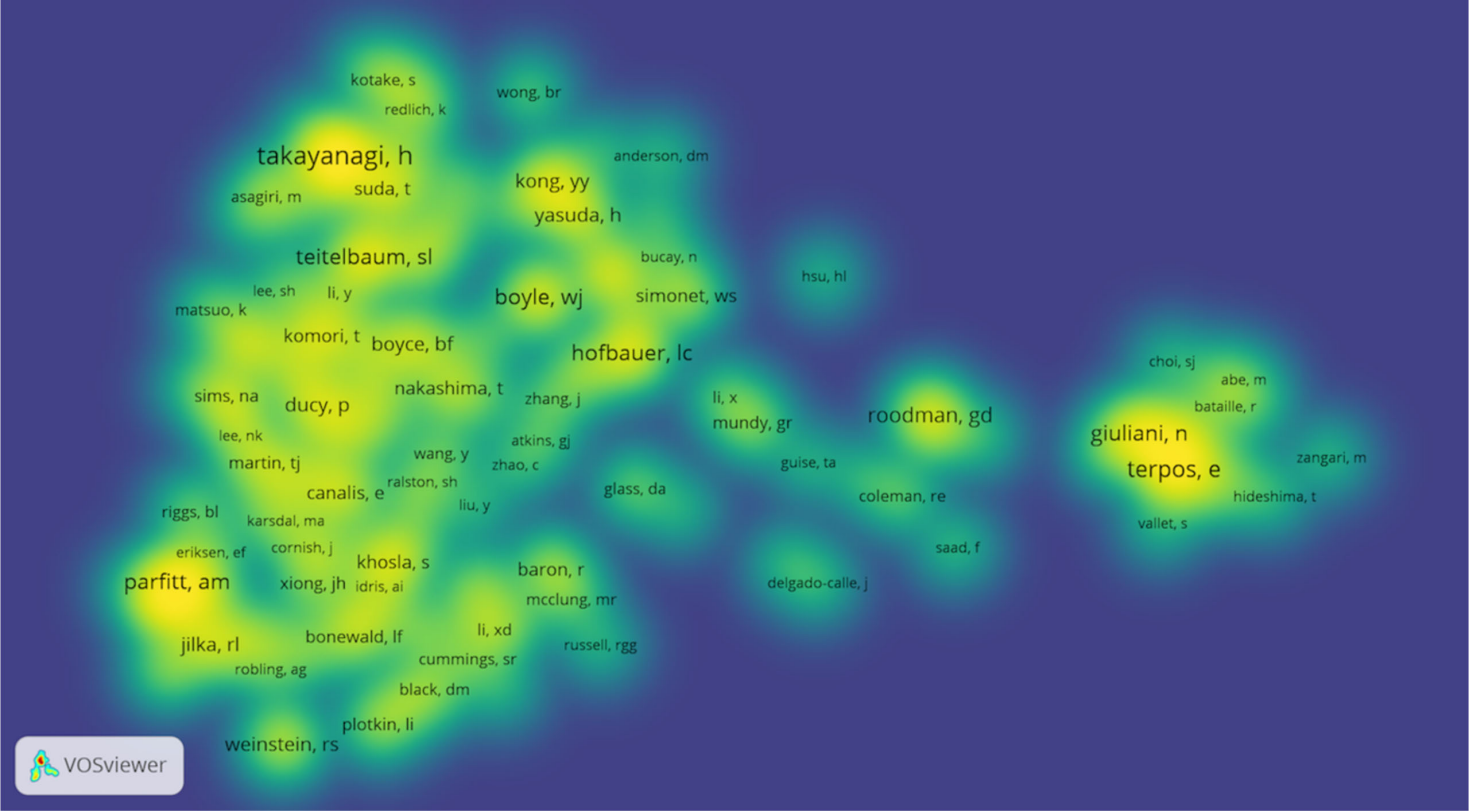
Density map of co-cited authors in osteoblasts-osteoclasts in bone disease (≥100). Notes: The size of the word and the opacity of yellow are positively related to the co-cited frequency.

VOSviewer software was used to analyze the authors and co-cited authors, and a total of 16,832 authors were found to have participated in studies related to osteoblasts-osteoclasts in bone disease, 58 of whom had published more than 10 papers (**Table 4**). Stavros C. Manolagas has the highest number of publications (n = 36), followed by Charles A. O’Brien (n = 29), Martina Rauner (n = 28), Maria Almida (n=27), David G. Roodman (n = 26), and Lorenz C. Hofbauer (n = 26). Authors with at least five publications (n = 340) are shown in the superposition visualization (**Figure 5**). As shown in the icons, different colors represent different years, and the same network colony represents active cooperation among different authors, such as network connection richest cluster authors: Stavros C. Manolagas and Charles A. O’Brien are followed by Martina Rauner and David G. Roodman, and yellow clusters JiaKe Xu and ShiWu Dong. Co-cited authors refer to those who are co-cited in different publications. The results indicated 69,589 co-cited authors, among which 883 were co-cited more than 30 times. The most frequently co-cited author is Hiroshi Takayanagi (n = 753), followed by Evangelos Terpos (n = 544), Steven L. Teitelbaum (n = 496), Michel A. Parfitt (n = 482), William J. Boyle (n = 466), Giuliani Nicola (n = 462), and Lorenz C. Hofbauer (n= 461). The remaining authors with co-citation ≥ 100 (n = 143) are presented on the density map (**Figure 6**). The density in the figure indicates a high frequency of the co-cited authors.

**Table 4:** The top 10 authors and co-cited authors of osteoblasts-osteoclasts in bone disease research.

Rank	Author	Count(%)	Co-Cited author	Co-citation	Centrality
1	Stavros C Manolagas	36(1.40%)	Takayanagi H	753	0.03
2	Chairles A O’Brien	29(1.10%)	Terpos E	544	0.03
3	Rauner Martina	28(1.10%)	Teitelbaum SL	496	0.00
4	Almeida Maria	27(1.00%)	Parfitt Am	482	0.01
5	Hofbauer Lorenz C	26(1.00%)	Boyle WJ	466	0.01
6	Roodman G David	26(1.00%)	Giuliani N	462	0.03
7	Xu Jiake	24(0.92%)	Hofbauer Ic	461	0.02
8	Weinstein Robert S	20(0.88%)	Manolagas SC	427	0.02
9	Jilka Robert I	20(0.88%)	Lacey dl	417	0.02
10	Ralston Stuart h	17(0.65%)	Roodman Gd	410	0.03

### Keyword Co-occurrence, Clusters, and Evolution

**Figure 7:**
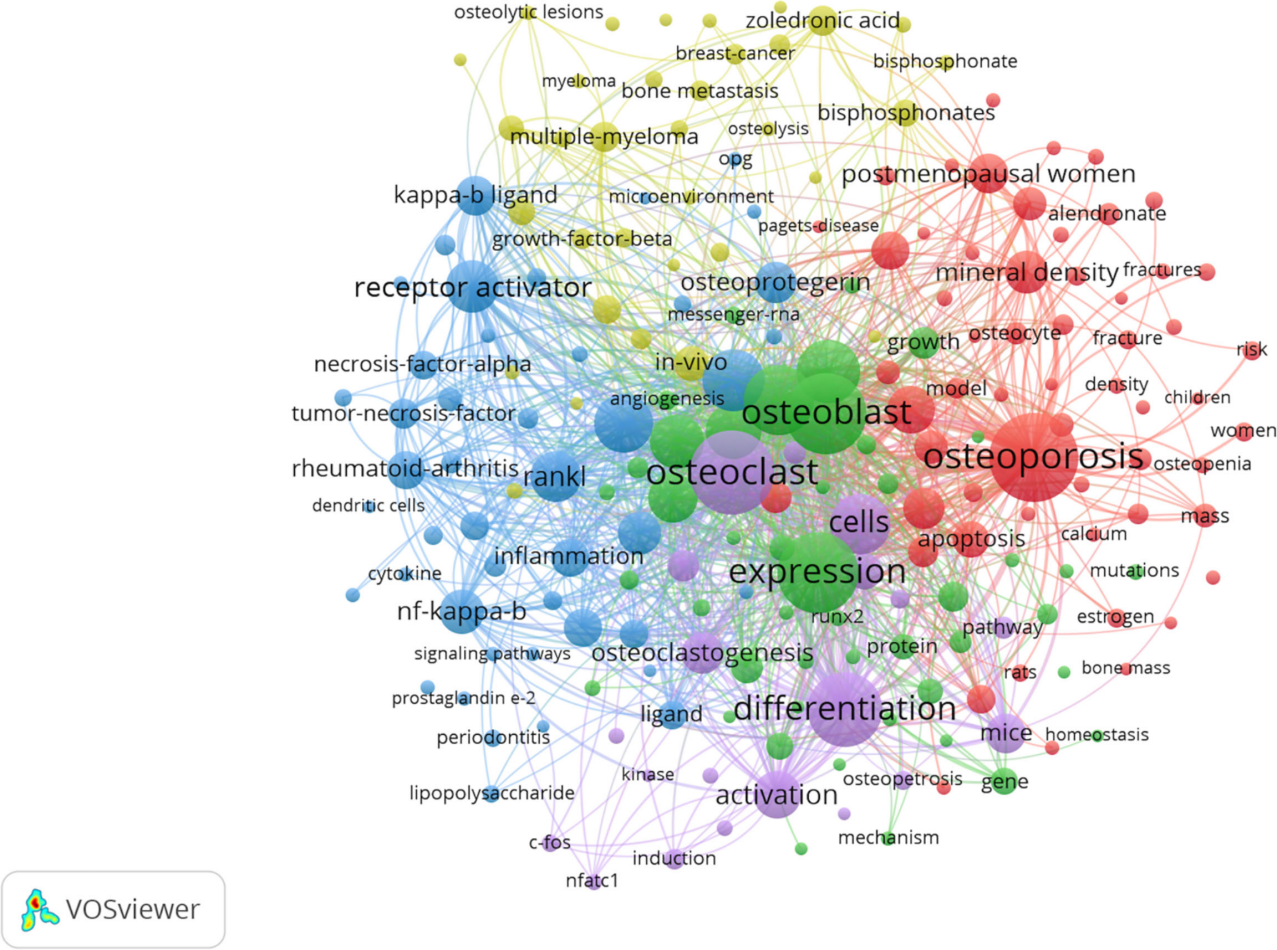
Keyword co-occurrence network and clusters in osteoblasts-osteoclasts in bone disease. Notes: The size of the node and word reflects the co-occurrence frequencies, the link indicates the co-occurrence relationship, and the same node color represents the same cluster.

Keyword co-occurrence analysis is the keyword analysis provided by the authors in the dataset. Keyword co-occurrence and emergent items were analyzed by CiteSpace and VOSviewer software. A total of 11,602 keywords were found by VOSviewer. Keywords related to osteoblasts-osteoclasts in bone diseases are presented in the network diagram (**Figure 7**). The top 10 keywords included osteoporosis (812), osteoclasts (743), expression (693), osteoblasts (691), differentiation (615), bone (459), receptor agonists (340), RANKL (310), mesenchymal stem cells (298), and diseases (278). Among all the keywords, 597 appeared more than 10 times and 119 appeared more than 50 times. As shown in **Figure 7**, osteoporosis was mentioned 812 times, and the frequency of the occurrence of corresponding keywords is shown in **Table 5**.

**Table 5:** The top 20 keywords involved in osteoblasts-osteoclasts in bone disease research.

Rank	Keywords	Count	Centrality	Rank	Keywords	Count	Centrality
1	Osteoporosis	812	0.04	11	Receptor Activator	340	0.01
2	Osteoclast	743	0.03	12	RANKL	310	0.01
3	Expression	693	0.02	13	Mesenchymal stem cells	298	0.04
4	Osteoblast	691	0.01	14	Activation	284	0.02
5	Differentiation	615	0.01	15	Disease	278	0.03
6	Bone	459	0.02	16	NF-KAPPA-B	254	0.04
7	Cell	423	0	17	Mineral Density	243	0.03
8	Osteoclast Differentiation	408	0.01	18	Gene-Expression	233	0.02
9	Osteoblasts Differentiation	364	0.01	19	Osteoprotegerin	229	0.02
10	*In Vitro*	347	0.02	20	Osteoclastogenesis	220	0.02

In the network analysis diagram, the keyword analysis was divided into five clusters with a high correlation within each cluster (**Figure 7**). The largest cluster, shown in red, contains a total of 58 terms, mainly related to osteoporosis. These include alendronate, apoptosis, association, autophagy, biochemical markers, bone formation, bone mass, bone metabolism, and bone metabolism mineral density. The second cluster, which is green, includes 46 terms and focuses on osteoblast differentiation. It also includes alkaline phosphatase, angiogenesis, beta-catenin, bone, bone regeneration, bone formation, bone marrow, and cartilage. The third cluster, the blue cluster, contains 45 terms related to osteoclast differentiation and includes activated protein-kinase, arthritis, bone loss, bone resorption, collagen-induced arthritis, colony-stimulating factor, cytokine, and dendritic cells. The fourth cluster, the yellow cluster, contains 34 terms, focusing on myeloma. In addition, it includes bisphosphonate, bone disease, bone metastasis, bone remodeling, breast cancer, cancer, denosumab, double-blind, etc. The fifth cluster is purple, with 19 terms covering molecular regulation mechanisms, including activation, induction, differentiation, osteoclast, and NFATC-1, c-Fos.

### Co-cite References and Reference Bursts

**Figure 8:**
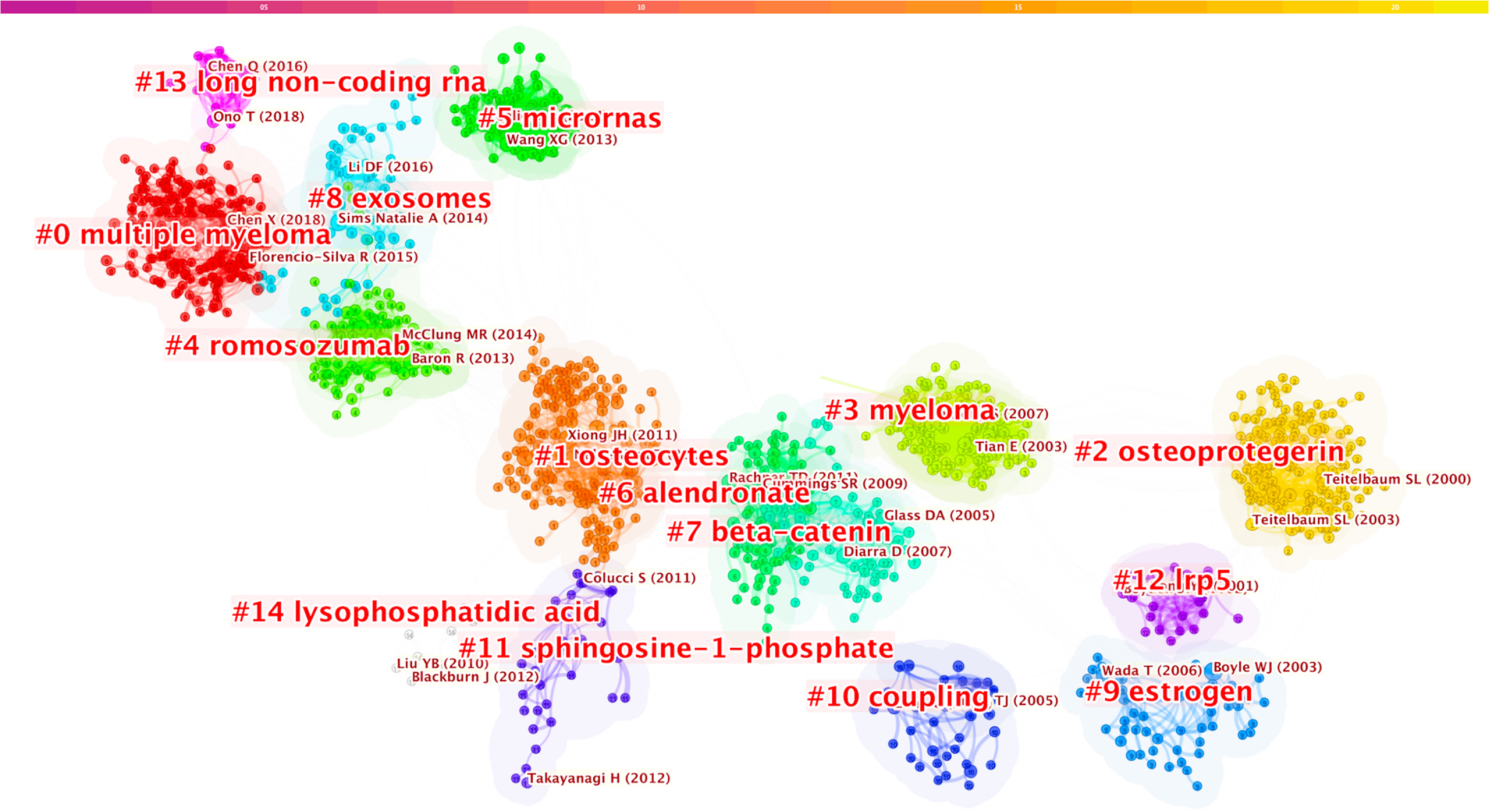
Keywords clustering of co-cited references in osteoblasts-osteoclasts in bone disease. Notes: The size of nodes represents the co-occurrence frequency of co-cited studies, and different colors represent different clustering of keywords.

Our analysis of the co-cited references found that the top 10 were co-cited more than 133 times, and three of these (William J. Boyle, 2003; Hiroshi Takayanagi, 2002; and Erming Tian, 2003) were co-cited more than 200 times (**Table 6**). The most co-cited reference is *“Osteoclast Differentiation and Activation”* published by William J. Boyle in *Nature* in 2003 (5). A keyword cluster analysis was conducted for co-cited references (**Figure 8**); different colors represent the clusters to which different co-cited studies belong, and the same colors indicate a close relationship between different co-cited studies. It is shown in the figure that keyword clustering divides co-cited studies into 15 categories. The top five clusters are multiple myeloma, osteoblast, osteocytes, myeloma, and romosozumab (an osteoblastic monoclonal antibody used to treat osteoporosis in postmenopausal women).

**Table 6:** The top 10 co-cited references for osteoblasts-osteoclasts in bone disease research.

Rank	Year	Reference	Journal	Co- Citation
1	2003	Osteoclasts differentiation and activation	Nature	463
2	2002	Induction and Activation of the Transcription Factor NFATc1(NFAT2) Integrate RANKL Signaling in Terminal Differentiation of Osteoclasts	Development Cell	222
3	2003	The role of the Wnt-Signaling Antagonist Dkk1 in the Development of Osteolytic Lesions in Multiple Myelola	The New England Journal of Medicine	165
4	2002	Evidence for osteocyte regulation of bone homeostasis through RANKL expression	Nature Medicine	164
5	2003	Genetic Regulation of Osteoclasts Development and Function	Nature reviews genetics	156
6	2002	The Novel Zinc Finger-Containing Transcription Factor Osterix is Required for Osteoblasts Differentiation and Bone Formation	Cell	149
7	2005	Canonical Wnt Signaling In Differentiation Osteoblasts Controls Osteoclasts Differentiation	Development Cell	148
8	2011	Matrix-embedded Cells Controls Osteoclasts Formation	Nature Medicine	133
9	2007	Osteoimmunology: Shared Mechanisms and Crosstalk Between the Immune and Bone Systems	Nature Reviews Immunology	125
10	2011	Osteoporosis: Now and The Future	Lancet	122

**Figure 9:**
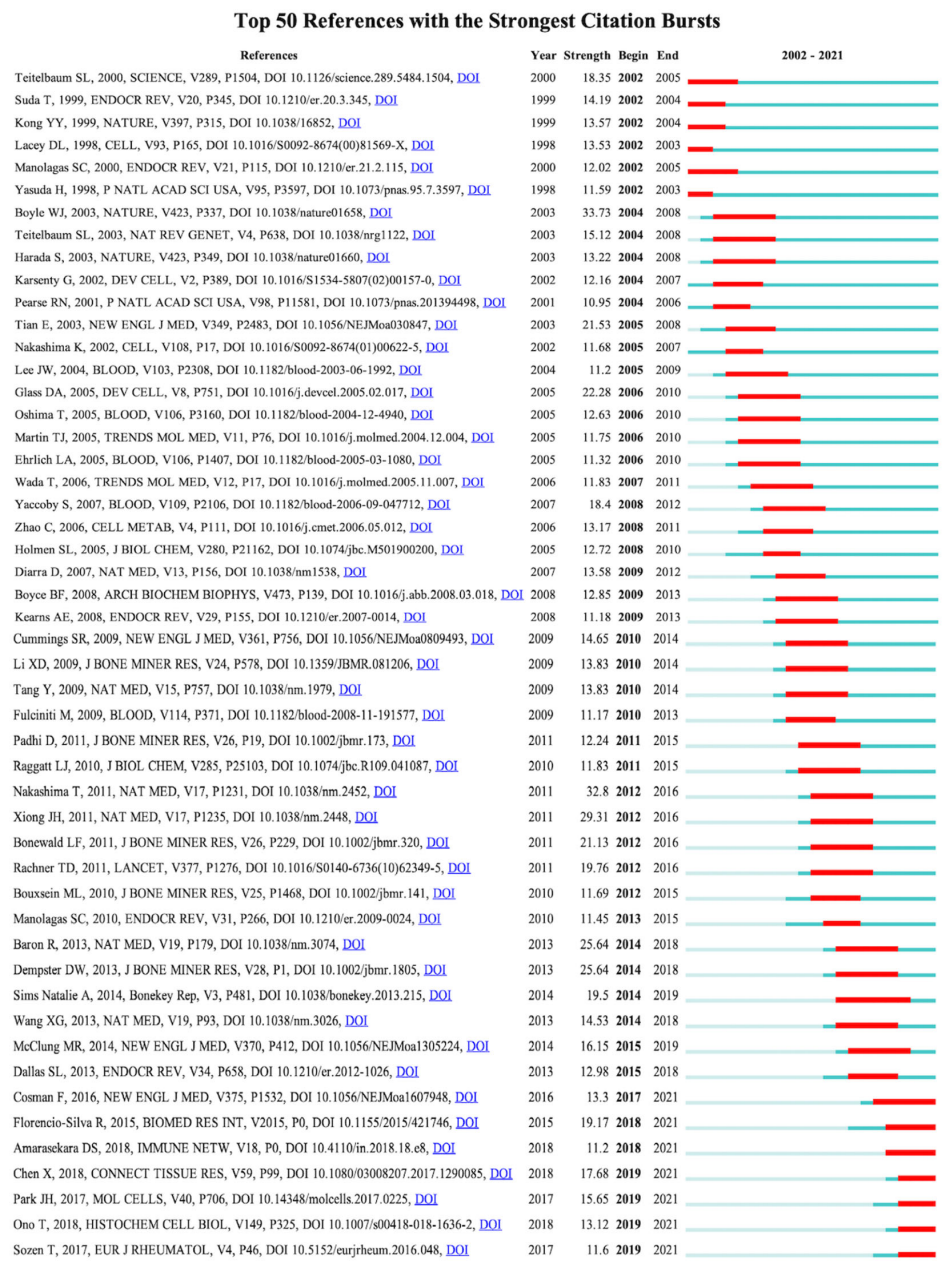
Top 50 references with the strongest citation bursts (sorted by the beginning year of burst). Notes: The blue bars mean the reference has been published; the red bars mean citation bursts.

Bursts analysis of CiteSpace co-cited reference was used, and the duration of bursts of co-cited studies was set to at least two years; the results showed that there were altogether 410 co-cited studies with citation bursts, and 50 studies with the strongest cited bursts were selected (**Figure 9**). **Figure 9** shows that 12% (6/50) of references in 2002 presented citation bursts, followed by 10% (5/50) in 2004 and 2012, and 8% (4/50) in 2006, 2010, 2014, and 2019. In 2004, *“Osteoclast Differentiation and Activation”* published in *Nature* by William J. Boyle was the most explosive (Strength = 33.73), which was consistent with the results of the co-citation literature analysis (5).

## Discussion

### General Information

The bone diseases related to osteoblasts-osteoclasts, including osteoporosis, osteosclerosis, Paget’s disease of bone, myeloma, and rheumatoid arthritis are common in older people. Moreover, approximately 40% of white women are affected by osteoporosis, and this number is expected to steadily increase in the future (6, 24). A total of 3,490 articles were retrieved from the Web of Science database from January 2002 to December 2021 on the topic of osteoblasts-osteoclasts in bone disease; in addition to the papers and review papers, 3,439 articles related to osteogenesis-osteoclasts in bone diseases were published in 928 journals, by 16,832 authors, by 579 institutions, and in 73 countries/regions. The number and trend of references published each year reflect the development speed and progress of a certain research field (25) (**Figure 2**). The number of publications has shown a trend of steady annual growth in the last 20 years, and the number of publications has increased by more than six times from 2002 to 2022.

Analysis of major and co-cited journals revealed that *Bone* published the most studies on osteoblasts-osteoclasts in bone diseases, whereas *Journal of Bone and Mineral Research* received the most co-citations (**Tables 1**, **2**). These two journals are related to the bone science-molecular mechanism, which is comparable to the double graph overlay analysis (**Figure 3**). A double graph overlay of journals shows the distribution of major journals. **Figure 3** shows the quotation of three main paths; one is from Molecular/Biology/Genetics co-cited journals to Molecular/Biology/Immunology journals, indicating that osteoblasts-osteoclasts in bone diseases research have focused on fundamental research.

The results of the CiteSpace analysis of osteoblasts-osteoclasts in bone diseases in different countries/regions and institutions also differ. **Table 3** and **Figure 4A** show that the US had the largest number of articles published in this field, with a total of 1,132 articles. In addition, the US was the first to conduct research on osteoblasts-osteoclasts in bone diseases as well as the largest contributor. This was followed by China, Japan, Germany, South Korea, Italy, and England (20, 26, 27). Five countries, the US, China, Germany, England, and Australia, present a high median centrality (≥ 0.1), which is considered a critical turning point leading to transformative discoveries. In addition, all of the top five countries except China are developed countries. In recent years, China has ranked second in the number of published articles, making a great contribution to this field, possibly because the number of published articles and research results are related to national researchers and financial support. The top 15 institutions are distributed in six countries, namely the US, China, Japan, Germany, South Korea, and Australia (**Figure 4B**). Among them, two-fifths are located in the US and one-fifth in China. The University of Arkansas for Medical Sciences, Sichuan University, Washington University, and Harvard University published the most articles and also actively cooperated with each other; thus, they all have made significant contributions to the field of osteoblasts-osteoclasts in bone diseases.

Considering authors and co-cited authors, Stavros C. Manolagas published the most articles, and his research on osteoblasts-osteoclasts in bone diseases was relatively early (**Table 4**). As shown in **Figure 5**, Stavros C. Manolagas, Charles A. O’Brien, and Piemontese Marlina have a close cooperative relationship. **Figure 6** shows that Hiroshi Takayanagi was co-cited the most times, and a cooperative relationship exists between different co-cited authors. The analysis of authors and co-cited authors in a certain field by VOSviewer software not only highlights influential researchers but also guides researchers by providing future directions and guidelines (28).

The intensity of co-citation bursts can also indicate emerging topics in the field. The high explosive intensity may indicate a turning point in the field. The top 10 co-citations were cited more than 100 times (**Table 6**). The largest eruption (33.73) corresponds to William J. Boyle et al.’s (2003) proposal on the mechanism by which the RANKL signaling pathway affects osteoclast formation and bone absorption activation because it provided insight into how hormonal signaling affects the bone structure (5). Since then, more pathways and regulatory mechanisms have been discovered, and RANKL signaling has become a new focus affecting osteoclast bone absorption. Subsequently, according to the explosive strength ranking, Tomoki Nakashima (2011) published in *Nature* ranked first (Strength=32.8), followed by Jinhui Xiong (2011) published in *Nature Medicine* (Strength=29.31). Both reported outbreaks that lasted for four years (2012–2016), indicating that RANKL signaling is an essential regulatory mechanism (26, 29). In third place is Roland Baron’s book published in *Nature* in 2013 (Strength=25.64), which also lasted for four years (2014–2018) and which illustrates the mechanisms of the Wnt signaling pathway in bone homeostasis (27).

### The Hotspots and Frontiers

Keyword co-occurrence and emergent item analysis can determine hot topics in a specific field in different periods and cluster keywords provided by authors in data sets (**Table 5**, **Figure 7**). In the research related to osteoblasts-osteoclast in bone diseases, the high-frequency keywords mainly include osteoporosis, osteoclast, expression, osteoblast, differentiation, bone, receptor agonist, RANKL, mesenchymal stem cell, and disease. These keywords are mostly located in the middle of the density map, which also reflects the research hotspots in this field. As illustrated by the keywords, this field mainly focuses on different signaling pathways and the functional effects (differentiation and expression) of receptors on osteogenic and osteoclast cells, leading to the occurrence of various bone diseases (30–34).

Keyword clustering describes the internal knowledge structure of a certain research field and classifies its domain (35). Cluster analysis showed that osteoblasts-osteoclast in bone diseases were divided into five categories (**Figure 7**), including osteoblasts-osteoclasts expression, osteoporosis, multiple myeloma, tumor necrosis factor, and exosome. Nowadays, osteoporosis and multiple myeloma have become major diseases worldwide, and the former, particularly, has an enormous impact on people’s health. Half of the women over 50 years old will experience fractures caused by osteoporosis (36). Studies on the molecular mechanism of osteoporosis and drug therapy have also attracted the extensive attention of a large number of researchers in recent years (6).

### The Role of Osteoclasts-Osteoblasts in Bone Disease

Osteoclast is a type of specialized cell the survival, proliferation, and differentiation of which are mainly regulated by the macrophage colony-stimulating factor, RANKL ligand, and osteoprotegerin (5, 31, 32). Osteoblasts are the main bone-forming cells; they produce a unique combination of extracellular proteins, including osteocalcin, alkaline phosphatase, and type I collagen (37). Bone homeostasis depends on the formation of bone by osteoblasts and the absorption of bone by osteoclasts; when the balance between the two is broken, the abnormal structure and function of bone will lead to the occurrence of various bone diseases. On the one hand, osteoblasts can undergo a variety of pathways influenced by osteoclast formation, differentiation, and apoptosis, such as RANKL/LGR4/RANK, Ephrin 2/EPHB4, and OPG/RANKL/RANK. On the other hand, osteoclasts can also influence the formation of osteoblasts by D2 isoform of vacuolar (H+) ATPase (v-ATPase) V0 domain (Atp6v0d2), Semaphorin 4D, complement component 3a, and microRNA (4, 38, 39).

RANKL/RANK signaling leads to the involvement of monocyte/macrophage progenitor cells in the activation of mature osteoclasts by regulating a variety of downstream signaling pathways. In the early stage of RANKL signaling, RANKL binds to RANK, enabling it to transmit intracellular signals by recruiting cohesion molecules to the cytoplasmic tail of the trimmer RANKL (e.g., tumor necrosis associated factor 6 (TRAF6)). Afterward, mitogen-activated protein kinases (MAPKs), transcription factor nuclear factor-kb (NF-κB), and activator protein-1 are activated (32, 40). Subsequently, T cell cytoplasmic 1 (NFATc1) nuclear factor is amplified and activated by the coordinated activation of AP-1 signal and co-stimulatory signal-mediated intracellular Ca2+ oscillation. Finally, activated NFATc1 transcribed osteoclast genes regulate the multinuclear and bone resorption functions of osteoclasts (35, 41, 42). However, OPG binds to RANKL and forms competitive inhibition with RANK to prevent excessive bone resorption (34, 43–45). Since the discovery that RANKL/RANK/OPG signaling pathway regulates bone resorption, it has provided further ideas for us to understand how bone construction and remodeling are regulated.

The Ephrin B family is composed of transmembrane proteins with cytoplasmic domains, whose interaction with EphB-expressing cells mediates bidirectional signal transduction (46). Zhaomei Chen et al. showed that osteoclasts express the NFATc1 target gene EfnB2 (encoding EphrinB2), whereas osteoblasts and other ephrin-Eph family members express the receptor EphB4 (38). They also demonstrated that the reverse signal of the bidirectional signal reaches the osteoclast precursor cell *via* EfnB2 and inhibits osteoclast differentiation by inhibiting the c-Fos–NFATc1 cascade of osteoclasts. Activated T cell cytoplasmic nuclear factor (NFATc1) is a transcription factor family originally found in T cells (47). In 2002, Hiroshi et al. demonstrated that NFATc1-deficient embryonic stem cells could not differentiate into osteoclasts under the stimulation of RANKL signaling, revealing that NFATc1 is the main regulator of osteoclast fusion, maturation, and activation (48). It regulates the migration and adhesion of osteoclasts to the bone surface through the induction of β3 Integrin (ITGB3) and c-Src. NFATc1 and PU.1 are involved in the regulation of β3 integrin expression during osteoclast differentiation (49). Subsequently, NFATc1 regulates osteoclast acidification and organic matrix degradation through ATP6i, CLC-7, and up-regulation of cathepsin K, MMP9, and LTBP3, respectively (50, 51). However, EfnB2-mediated EphB4 signaling to osteoblasts during forward signal transduction (osteoclast to osteoblast) promotes osteoblast differentiation, thus inhibiting apoptosis. These results demonstrate that the ephrin-Eph bidirectional signaling pathway connects two major molecular mechanisms in osteoblast and osteoclast to maintain bone homeostasis (46, 52).

Increasing evidence indicates that multiple microRNAs are important regulatory factors of bone formation genes (53). Wang et al. demonstrated experimentally that the transfer of an osteoclast-derived exosome miR-214 to osteoblasts directly targets ATF4 to inhibit osteoclast activity, whereas antagomiR-214 promotes osteoblast activity and matrix mineralization *in vitro* and *in vivo (* 54, 55). Yosuke et al. found that miR-125b inhibited osteoblast proliferation and differentiation as well as the ERBB2 receptor tyrosine kinase (56). Hiroyuki et al. showed that miR-206 was expressed in osteoblasts, but its expression decreased during osteoblast differentiation, and its overexpression inhibited osteoblast differentiation (57). Yosuke et al. found that miR-210 promoted the osteoblasts of ST2 cells by transfecting miR-210 in ST2 cells and verified that miR-210 could inhibit the TGF-β/actin signaling pathway by targeting ACVR1B to promote osteoblast differentiation (58).

### Osteoblasts-Osteoclasts Research in Tropical Bone Diseases

Using VOSviewer and CiteSpce to analyze the keywords, we found that the top three bone diseases were osteoporosis, rheumatoid arthritis, and multiple myeloma. In addition, VOSviewer co-operative network analysis of keywords was used to generate five large clusters, among which the largest was osteoporosis (**Figure 7**). When using CiteSpce for keyword clustering analysis of literature, the results showed that the top cluster was multiple myeloma (**Figure 8**). Thus, we describe the important role of osteogenesis-osteoclasts in the top three bone disorders.

Due to the aging population, osteoporosis has become a major health concern globally. In 2016, the National Osteoporosis Foundation (NOF) reported that one-half of women, especially postmenopausal women, and as many as one-quarter of men over the age of 50 develop fractures due to osteoporosis. Human bone is always in a dynamic balance between bone formation and bone resorption. When bone resorption is greater than bone formation, the bone structure is destroyed, the ratio of bone mineral composition to bone matrix per unit volume decreases, and bone fragility increases while bone density decreases, which can lead to osteoporosis (59, 60). Primary osteoporosis is divided into Type I and Type II; the former is mainly distributed among women shortly after menopause, and the latter mainly in patients older than 75. The main cause of primary osteoporosis is bone loss resulting from the osteoblasts’ reduced bone formation ability. In recent years, the treatment of osteoporosis has also received considerable attention. Denosumab, an all-human monoclonal antibody against nuclear factor-κB ligand-receptor activator, blocks the binding of RANKL to RANK, inhibits osteoclast development and activity, decreases bone resorption, and increases bone mineral density. Steven R. Cummings et al. demonstrated the potential of denosumab to provide an alternative approach to the treatment of osteoporosis (61–64). Romosozumab has also been proved effective in the treatment of osteoporosis because it not only rapidly improves bone mineral density and significantly reduces the risk of fracture, but also significantly increases bone formation markers and bone mineral density of the spine and hips, compared to alendronate or teriparatide (65).

Rheumatoid arthritis (RA) is a chronic inflammatory autoimmune disease mainly characterized by arthropathy, affecting approximately 1% of the population. Its high-risk factors include age, gender, genetics, and environment (e.g., smoking, air pollution, and occupation), and the incidence of RA in females is much higher than that in males (66–68). Studies have shown that osteoclasts in RA have better bone resorption than osteoblasts in bone formation and occupy the interface between inflammatory synovial tissue and bone surface around the joint; thus, osteoclasts are the key to joint destruction in RA (7). This joint inflammation is initiated and maintained by complex interactions between different dendritic cell (DC) subtypes, T cells, macrophages, B cells, neutrophils, fibroblasts, and osteoclasts (69, 70). At the same time, cytokines such as tumor necrosis factor α (TNF) and interleukin 6 (IL-6) play a special role in the inflammatory process of RA (71). It has been reported that inhibition of osteoclast differentiation by inhibiting RANKL or macrophage colony-stimulating factor (M-CSF) plus RANKL prevents bone erosion in animal models of arthritis (7, 72, 73). In addition, direct targeting of osteoclasts in RA also protects bone from inflammation (74, 75). Considering the Janus family of kinases (JAKs), activation of JAK/STAT signals through JAK mutations or abnormal TYK2 signals constituted by JAK is crucial for inducing autoimmunity and some immune deficiency syndromes (76, 77). At present, the drugs used to treat RA mainly aim at controlling pain and inflammation and reducing joint injury, while the development of disease-modifying antirheumatic drugs (DMARDs) has made considerable progress. Existing DMARDs can be divided into three categories, namely conventional synthetic DMARDs (methotrexate, sulfadiazine, and hydrochloroquine), targeted synthesis of DMARDs (pan-JAK- and JAK1/2-inhibitors), and biosynthesis of DMARDs (tumor necrosis factor (TNF)-α inhibitors, anti-interleukin ‐6‐receptor monoclonal antibodies (mABs), JAK inhibitors, and B cell depleting antibodies) (5, 78–81).

Multiple myeloma (MM) ranks first in the co-cited references (**Figure 8**), analyzed co-cited references, and conducted keywords macro category by CiteSpace. The results indicate that it has become a research hotspot in the field of osteoblasts-osteoclasts in bone disease in recent years. MM is a terminally differentiated plasma cell tumor that grows in the bone marrow and is associated with osteolysis bone disease, mainly caused by the imbalance of bone remodeling resulting from the increased osteoclast activity and decreased osteoblast activity, as well as changes in number and function (82). The age-adjusted incidence is approximately 4/100,000, accounting for 10% of all malignancies (83). Dickkopf Wnt signaling pathway inhibitor 1 (DKK1) is a secretory factor related to the function of osteoblasts and also a soluble Wnt signaling inhibitor. Myeloma cells contain detectable DKK1, which obstructs the differentiation of osteoblasts, promotes the proliferation of osteoblasts, changes the balance between osteoblasts and osteoclasts, and develops into osteolytic lesions. The antagonistic activity of DKK1 in myeloma can reduce osteolytic absorption, which is beneficial for controlling the growth of multiple myeloma (82, 84–86). Wnt family signaling is a large family of growth factors that mediate basic biological processes such as embryo, organ, and tumorigenesis, and plays an important role in the growth and differentiation of osteoblasts (84, 87, 88). Increasing evidence indicates that an increase in the Wnt signaling pathway in a bone micro-environment can prevent the occurrence of myeloma, inhibiting the growth of myeloma in the bone by antagonizing DKK1 and DKK3 (89, 90). These would be potential therapeutic targets for MM.

In summary, Osteoporosis, Rheumatoid Arthritis (RA), and multiple myeloma have been the top 3 diseases as hot topics for research. Researchers have focused on the pathogenesis of Osteoblasts-osteoclasts in bone diseases and some drugs have been used for clinical treatment. In the future, more bone disease targets are expected to be discovered and more targeted drugs will be developed by researchers. and the application of advanced technologies on clinical and osteoblast-osteoclast-related bone diseases should be investigated further.

## Conclusion

Osteoblasts-osteoclasts in bone diseases have been of great concern to researchers, and countries/regions and institutions worldwide have actively cooperated with each other in their research, with the US as the main cooperation center. Current publications focus on molecular biology and clinical medicine. The study of osteoblasts-osteoclasts in bone diseases mainly includes four aspects: the regulatory mechanism, osteoblast-osteoclast differentiation, osteoporosis, and bone tumor. Current research focuses on oxidative stress, mutation, osteoblast formation, absorption, bone metabolism, tumor therapy, and in-depth mechanisms.

## Data Availability Statement

The raw data supporting the conclusions of this article will be made available by the authors, without undue reservation.

## Author Contributions

QH, XN contributed to the conception of the study; JH, and HS performed the experiment and contributed significantly to the analysis and manuscript preparation; WQ, KL, SJY performed the data analyses and helped write the manuscript; KP, BZ, SHY, XP helped perform the analysis with constructive discussions. All authors contributed to the article and approved the submitted version.

## Funding

This study was supported by grants from the Guangxi Key Research and Development Plan(2021AB11027);Clinical research climbing plan of the First Affiliated Hospital of Guangxi Medical University(YYZS2020010);Key Research and Development Plan of Qingxiu District, Nanning City(2020053)

## Conflict of Interest

The authors declare that the research was conducted in the absence of any commercial or financial relationships that could be construed as a potential conflict of interest.

## Publisher’s Note

All claims expressed in this article are solely those of the authors and do not necessarily represent those of their affiliated organizations, or those of the publisher, the editors and the reviewers. Any product that may be evaluated in this article, or claim that may be made by its manufacturer, is not guaranteed or endorsed by the publisher.
